# Endoscopic characterization of oropharyngeal dysphagia in patients with dementia

**DOI:** 10.3389/fragi.2025.1535137

**Published:** 2025-06-26

**Authors:** Sara Peranovic, Maryam Pourhassan, Bendix Labeit, Paul Muhle, Sonja Suntrup-Krueger, Tobias Warnecke, Rainer Dziewas, Ulrike Trampisch, Rainer Wirth, Gero Lueg

**Affiliations:** ^1^ Department of Geriatric Medicine, Marien Hospital Herne, Ruhr University Bochum, Herne, Germany; ^2^ Department of Neurology, University Hospital Düsseldorf, Düsseldorf, Germany; ^3^ Department of Neurology, University Hospital Münster, Münster, Germany; ^4^ Department of Neurology and Neurorehabilitation, Klinikum Osnabrück, Osnabrück, Germany

**Keywords:** dysphagia (swallowing disorder), dementia, alzheimer’s diaease, Parkinson’s disease dementia, fees, geriatric patients

## Abstract

**Objective:**

Diagnosing and treating dysphagia in patients with dementia is challenging and few studies have been performed to characterize dysphagia based on Flexible Endoscopic Evaluation of Swallowing (FEES). Therefore, we aimed to characterize and compare the dysphagia pathologies in various stages and types of dementia.

**Methods:**

This is a retrospective study of 107 hospitalized geriatric patients with dysphagia and Alzheimer’s dementia, Alzheimer’s dementia with moderate to severe cerebral vasculopathy (mixed dementia), and patients with dementia associated with Parkinson’s syndrome who underwent FEES. A standardized FEES protocol was used to characterize the dysphagia pathologies, including premature bolus spillage, delayed swallowing reflex and bolus residue as well as penetration and aspiration and the white-out intensity. The distribution of different dysphagia pathologies was cross-tabulated with χ2 statistics across different types of dementia.

**Results:**

A comparative analysis of dysphagia pathologies across the three dementia types revealed a relatively mixed picture of various dysphagia findings in all dementia types. However, a significantly higher prevalence of bolus penetration and complex dysphagia, which was defined as presence of at least two major findings simultaneously within a patient, was seen in patients with Parkinson’s-related dementia compared to other forms of dementia. In general, residue was the most frequent finding in all types of dementia (78%–100%). In contrast, aspiration was the least prevalent finding with no significant variation between dementia types.

**Conclusion:**

Although participants with Parkinson’s-related dementia exhibited minor specific findings, our study revealed no distinct endoscopic dysphagia pathologies across various types of dementia.

## Introduction

Oropharyngeal dysphagia (OD) is recognized as a critical geriatric syndrome that significantly diminishes the quality of life in older patients and is associated with several adverse outcomes, including hospitalizations, pneumonia, malnutrition, dehydration and increased mortality (Baijens et al., 2016; Banda et al., 2022).

The etiology of OD is complex and depends primarily on the presence of neurological diseases and age-related changes in multiple swallowing domains including sensory function and disease related changes of anatomical structures (Rajati et al., 2022; Abu-Ghanem et al., 2020). One of the neurological diseases that is frequently associated with dysphagia is dementia. Contrary to extensive research that has been conducted on dysphagia in stroke patients, where dysphagia may diminish over days to weeks, less is known about the dynamic in neurodegenerative diseases that typically show a progressive decline (Parlak et al., 2022; Sheikhany et al., 2019). As the condition advances in patients with dementia, there is often a notable decrease in pharyngeal clearance and opening of the upper esophageal sphincter, accompanied by frequent occurrences of penetration and/or aspiration (De Stefano et al., 2020; Mira et al., 2022). These changes exacerbate the risk of severe health consequences, underscoring the need for precise diagnostic and management strategies.

Flexible endoscopic examination of swallowing (FEES) is a well-established, reliable, bedside diagnostic tool for assessing swallowing disorders in geriatric patients (Dziewas et al., 2024). Recent advancements include FEES-based classification of dysphagia phenotypes (Warnecke et al., 2021). In recent studies, the use of FEES mainly focussed on geriatric patients with cognitive impairment resulting from cerebrovascular accidents and pneumonia (Benjapornlert et al., 2020), presbyphagia (Labeit et al., 2022) or rare neurodegenerative diseases such as amyotrophic lateral sclerosis (Tye et al., 2021). In particular, characteristic dysphagia patterns have been associated with Parkinson’s disease, stroke and neuromuscular diseases (Warnecke et al., 2021). Nevertheless, the specific swallowing pathologies associated with Alzheimer’s disease and other forms of dementia remain to be established, and the impact of dementia on dysphagia is still poorly understood. Accordingly, the objective of the present study was to provide a systematic analysis of the endoscopic pathologies associated with different types of dementia and concomitant dysphagia.

## Methods

### Subjects and methods

This retrospective analysis was conducted in a cohort of inpatients with dementia who underwent FEES between January 2019 and January 2024 on a geriatric ward. The most common reasons for performing FEES were weight loss of more than 3 kg within 3 months and/or a total score of ≤7 on the Mini Nutritional Assessment Short-Form (MNA-SF) (n = 47), indicating relevant malnutrition. Other reasons included abnormalities detected during speech therapist examinations (e.g., wet voice after swallowing water, dysarthria, and reduced cough force; n = 32), symptoms reported by caregivers (e.g., reduced appetite; n = 18), a history of dysphagia (n = 17), and self-reported swallowing difficulties (n = 13). In some cases, aspiration pneumonia was documented in the medical history (n = 11), and buccofacial apraxia was present (n = 5). Ten patients reported no prior symptoms. In patients with severe cognitive impairment, FEES was performed with the support of trained geriatric nurses and, when appropriate, in the presence of a familiar caregiver. The examination was carried out in a quiet setting with simplified instructions, and was immediately discontinued if distress occurred.

The findings were evaluated by two certified examiners with significant experience in the field of neurogenic dysphagia. The videos and findings were retrospectively reviewed by the first author and the findings were reevaluated. In case of deviations from the previous findings, a certified FEES specialist was consulted. The interrater reliability showed a strong agreement (kappa = 0.84). Patients were included if they were >65 years with either a pre-existing or newly diagnosed dementia and if a FEES-video for analysis was available. In the case of Parkinson’s-related dementia, Parkinson’s disease was diagnosed according to the Movement-Disorder-Society Criteria and graded according to the modified Hoehn and Yahr scale (Postuma et al., 2015; Goetz et al., 2004). The diagnosis of Alzheimer’s disease was made in accordance with the established diagnostic criteria outlined in the DSM-5 (Simpson, 2014). The diagnosis was based on a comprehensive clinical assessment, neuropsychological testing, and structural cerebral imaging (MRI or CT). When available, biomarker data (e.g., cerebrospinal fluid analysis) were incorporated. However, due to the retrospective and clinical nature of the study, the availability of biomarker testing was not uniform. Consequently, patients were also included if the diagnosis of Alzheimer’s disease was made based on the clinical syndrome, disease course, and supporting imaging findings. Clinical presentation and neurocognitive testing were used to classify the severity of dementia (Nasreddine et al., 2005; Williams et al., 2013). The diagnosis of “mixed dementia” was assigned to patients with Alzheimer’s dementia who exhibited cerebral microangiopathy with a Fazekas score of ≥2, as determined by MRI findings. In cases where MRI findings were not available, the diagnosis was based on CT (Scheltens et al., 1998). Patients with history of stroke other than lacunary infarction were excluded. Additional exclusion criteria included an incomplete data set and the presence of delirium as a potential cause of cognitive impairment. The study protocol was approved by the Ethics Committee of the Ruhr University Bochum (Reg.-Nr. 23–7,878). The study is registered at German Clinical trial register (DRKS-ID: DRKS00032433).

### Geriatric assessment

All study participants underwent a comprehensive geriatric assessment. Cognitive capabilities were evaluated using the Montreal Cognitive Assessment (MoCA) within the first 48 h of admission (Nasreddine et al., 2005). For patients with significant hearing impairment, the auditory subtests of the MoCA (digit span, sentence repetition and, if applicable, cued recall) were excluded. To ensure comparability with the standard 30-point scale, the maximum possible score was proportionally adjusted and the total score extrapolated. Similarly, patients with functional blindness received the MoCA-Blind version, which omits vision-dependent tasks such as trail-making, cube copying, clock drawing, and naming. This version has a maximum score of 22 points. Scores obtained were interpreted using established normative data for the MoCA-blind (Rossetti et al., 2011). Nutritional status was assessed using the MNA-SF (Kaiser et al., 2009), categorizing individuals into normal nutritional status (12–14 points), at risk of malnutrition (8–11 points), and malnourished (0–7 points). Performance of activities of daily living was assessed using the German version of the Barthel Index (Fi, 1965), where a scale from 0 to 100 reflects varying degrees of independence with higher scores denoting greater independence. Frailty was assessed with the Clinical Frailty Scale, which combines descriptive texts and pictograms to illustrate a patient’s functional and activity level, rated from 1 (very fit) to 9 (terminally ill) (Sternberg et al., 2011). Sarcopenia was assessed using the SARC-F questionnaire, with scores greater than 4 indicating probable sarcopenia (Drey et al., 2020). Handgrip strength was measured three times on the dominant or unaffected side, with the highest value recorded (Cruz-Jentoft et al., 2019).

### Assessment of dysphagia

FEES and the subsequent evaluation of the video were performed collaboratively by a speech-and-language therapist and a physician, both of whom with extensive experience in the field of neurogenic dysphagia. The assessments utilized an ENF-VH2 laryngoscope provide by Olympus (Hamburg, Germany) and a video documentation system from Rheder/Partner (Hamburg, Germany) following a standardized protocol. In addition to assessing anatomical structures and evaluation of saliva management, different food consistencies were tested to examine swallowing function comprehensively in accordance with a well-established protocol (Dziewas et al., 2024) in the following order: Green coloured jelly (IDDSI-level 4), nectar-like thickened (IDDSI-level 2) and non-thickened green coloured water (IDDSI-level 0), and three trials of white solid bread measuring approximately 3 cm × 3 cm × 0.5 cm (Cichero et al., 2017). For each food consistency the occurrence of premature spillage, residue, penetration, aspiration was documented. The white-out, a crucial observational landmark where the pharyngeal wall undergoes maximal contraction resulting in white superimposition from light reflection of the endoscope, was specifically categorized as “complete” or “incomplete” at the first swallow of every bolus consistency based on a visual scale (Labeit et al., 2021a). Since the Penetration Aspiration Scale (PAS) (Rosenbek et al., 1996) very much focusses in airway safety and does not evaluate premature bolus spillage and residue, dysphagia severity was additionally categorized according to a FEES dysphagia severity score with four different levels of severity (Warnecke et al., 2016): Grade 0 denoted no clinically relevant neurogenic dysphagia, grade 1 represented the mildest form of neurogenic dysphagia (premature spillage and/or significant residue, but no penetration or aspiration), grade 2 indicated moderate neurogenic dysphagia with penetration or aspiration of one food consistency, and grade 3 indicated severe neurogenic dysphagia with penetration or aspiration of two or more food consistencies.

We used the following parameters for the characterization of swallowing disorders:

• Premature bolus spillage: As in a previous study classifying different swallowing patterns in neurological disorders, premature spillage before triggering the swallowing reflex was defined as occurring at the level of the piriform sinus (Warnecke et al., 2021).
• Delayed swallowing reflex: As in previous studies, delayed swallowing was defined as a delay of at least 3 seconds after the bolus reached the vallecula without triggering a swallowing reflex (Warnecke et al., 2021; Labeit et al., 2021b).
• Residue in the valleculae and residue in the piriform sinus: All remaining except for coating after the first swallow was evaluated according to the Yale-Scale (Neubauer et al., 2015). Bolus residues were evaluated for each swallow and tested consistency. For the analysis, the worst residue finding across the three swallows for each consistency was used. The location of the residue (valleculae versus piriform sinus) was documented separately.
• Penetration and Aspiration were rated according to the PAS (Rosenbek et al., 1996).
• White-out was rated as “incomplete”, when the frame with the maximum white superimposition was not completely white (Labeit et al., 2021a).
• Complex swallowing disorder was rated if at least two of the abovementioned mechanisms occurred simultaneously within one patient. For better illustration, the localization of the residue depending on their localization in the vallecular space or in the piriform sinus is shown in Table 3. When defining a complex dysphagia pathology, the localization of the residues was not considered separately.

### Statistical analysis

Statistical analyses were conducted using SPSS software (SPSS Statistics for Windows, Version 29.0, IBM Corp., Armonk, NY, USA). Continuous variables were reported as means ± standard deviations (SDs) for normally distributed data and medians with interquartile ranges (IQRs) for non-normally distributed data. Categorical variables were presented as counts and percentages. The distribution of dysphagia pathologies across different types of dementia was assessed using cross-tabulation, comparing observed counts against expected counts derived from χ2 statistics. For each type of dementia where n ≥ 5, a χ2 test of independence was utilized to examine the relationship between each dysphagia pathology and the type of dementia. When expected counts were less than 5, Fisher’s exact test was applied instead. The prevalence of dysphagia pathologies across different dementia types was also visualized in a bar chart. A regression analysis was performed to explore the independent effects of various risk factors—including age, gender, dementia severity, sarcopenia, handgrip strength, Barthel Index, and frailty—on the severity of dysphagia. Additionally, partial correlation analyses were conducted to assess the associations between dysphagia pathologies. A p-value of <0.05 was considered statistically significant.

## Results

Baseline characteristics of study participants are presented in Table 1. Of the 313 geriatric inpatients with dementia who were screened between 2019 and 2024, 107 patients with endoscopically confirmed oropharyngeal dysphagia were included in the final analysis. Reasons for exclusion included missing or incomplete FEES (n = 87), predefined exclusion criteria such as delirium or infection (n = 60), and an absence of pathological FEES findings (n = 59). A detailed flowchart of patient selection is provided in Supplementary Figure S1. The age of the study population ranged between 65 and 96 years (82.5 ± 6.7 years), with 54% being females. Major reasons for hospital admission included falls and fractures, immobility after surgery cardiovascular diseases, malnutrition, heart failure and neurodegenerative diseases. The Barthel index recorded a median score of 40, indicating a moderate to severe dependency in activities of daily living among the participants. Furthermore, 80 patients (74.8%) were identified as having probable sarcopenia according to the Sarc-F score and frailty was observed in 97 patients (91.5%) based on Clinical Frailty Scale.

**TABLE 1:** Characteristics of the study population on admission.

Parameter	Total population (n=107)
Gender (n, %)	
Females	49 (54)
Males	58 (46)
Age (y)	82.5 ± 6.7
Hand grip strength (kg)	15.1 ± 9.7
Geriatric assessment, Median (IQR)	
Barthel-Index	40 (25-45)
Clinical Frailty Scale	6 (6-7)
SARC‐F scores MNA-SF	8 (7-9) 7 (2-14)
Cognitive function (MoCA) Hoehn and Yahr scale	14 (9-17) 4 (2–5)

Values are given as number (%), mean ± SD, or median (IQR, interquartile range). Mini Nutritional Assessment short-form (MNA-SF); Montreal Cognitive Assessment (MoCA).

Table 2 summarizes the prevalence and severity of different dementia types and dysphagia pathologies as assessed by FEES. The cohort comprised 107 patients, with one-third of each dementia type including Alzheimer’s disease, mixed-type dementia, i.e., Alzheimer’s disease with vascular lesions and Parkinson’s-related, i.e., Parkinson’s disease with dementia and or possible diagnosis of Lewy body dementia (McKeith et al., 2017). Notable dysphagia pathologies included a high prevalence of residue in valleculae (96%), impaired white out (75%), and a complex dysphagia pathology (72%), with aspiration being the least common condition observed in 13%.

**TABLE 2:** Prevalence of dementia type and dysphagia pathologies among study participants.

Parameter	Total population (n = 107)
Dementia (n, %)	
Yes	107 (100)
Dementia severity (n, %)	
Mild	43 (40)
Moderate	40 (38)
Severe	24 (22)
Dementia type (n, %)	
Alzheimer-dementia	32 (30)
Parkinson’s-related dementia	38 (35)
Mixed type dementia	37 (35)
Dysphagia severity (n, %)	
Mild	52 (49)
Moderate	37 (34)
Severe	18 (17)
Dysphagia phenotype (n, %)	
Premature bolus spillage	
Yes	36 (34)
No	71 (66)
Delayed swallowing reflex	
Yes	25 (23)
No	82 (77)
Residue valleculae	
Yes	103 (96)
No	4 (4)
Residue piriform sinus	
Yes	85 (79)
No	22 (21)
Penetration	
Yes	48 (45)
No	59 (55)
Aspiration	
Yes	14 (13)
No	93 (87)
Impaired white out	
Yes	80 (75)
No	27 (25)
Complex	
Yes	77 (72)
No	30 (28)

**FIGURE 1:**
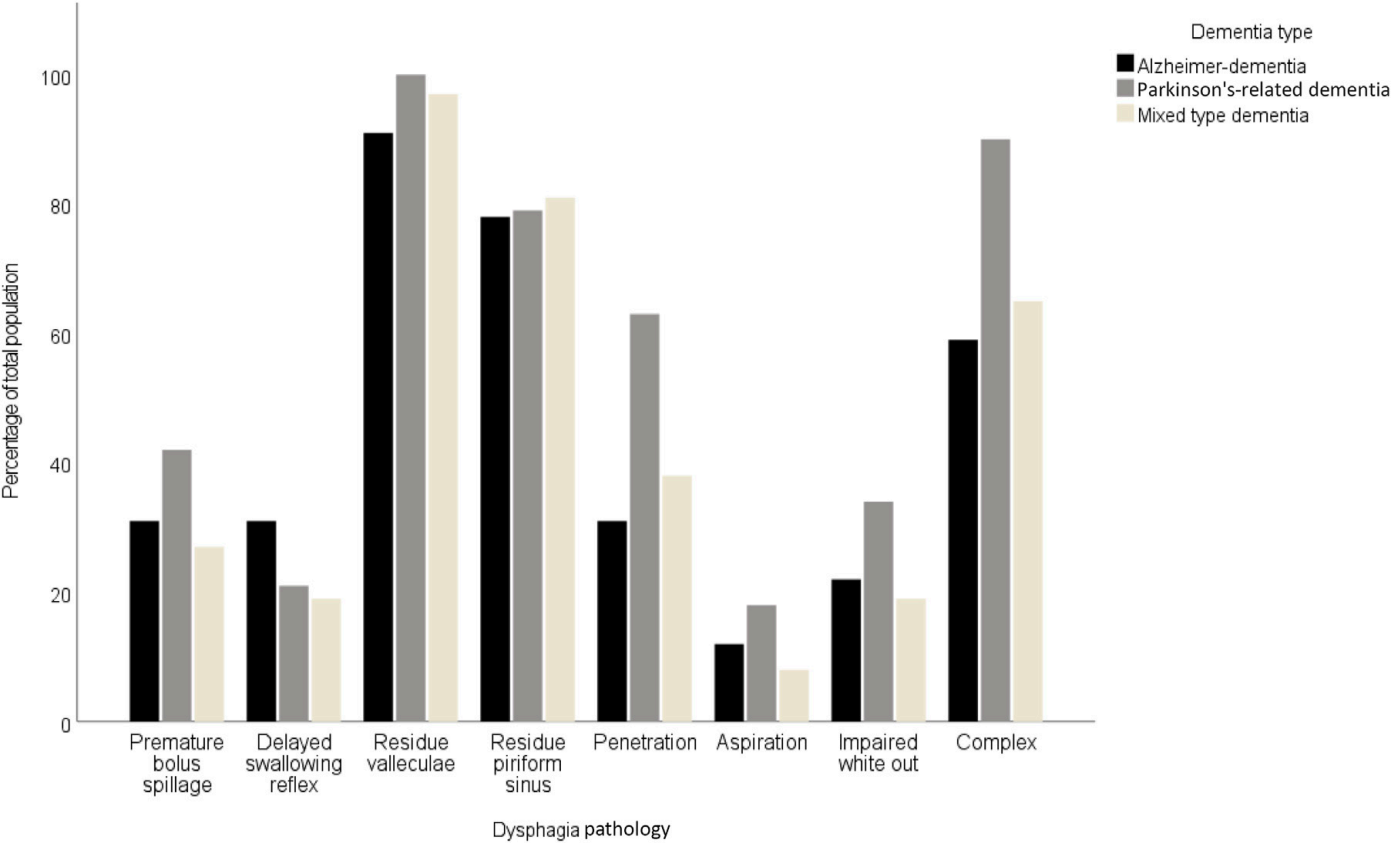
Distribution of dysphagia pathologies in patients with different types of dementia.

**FIGURE 2:**
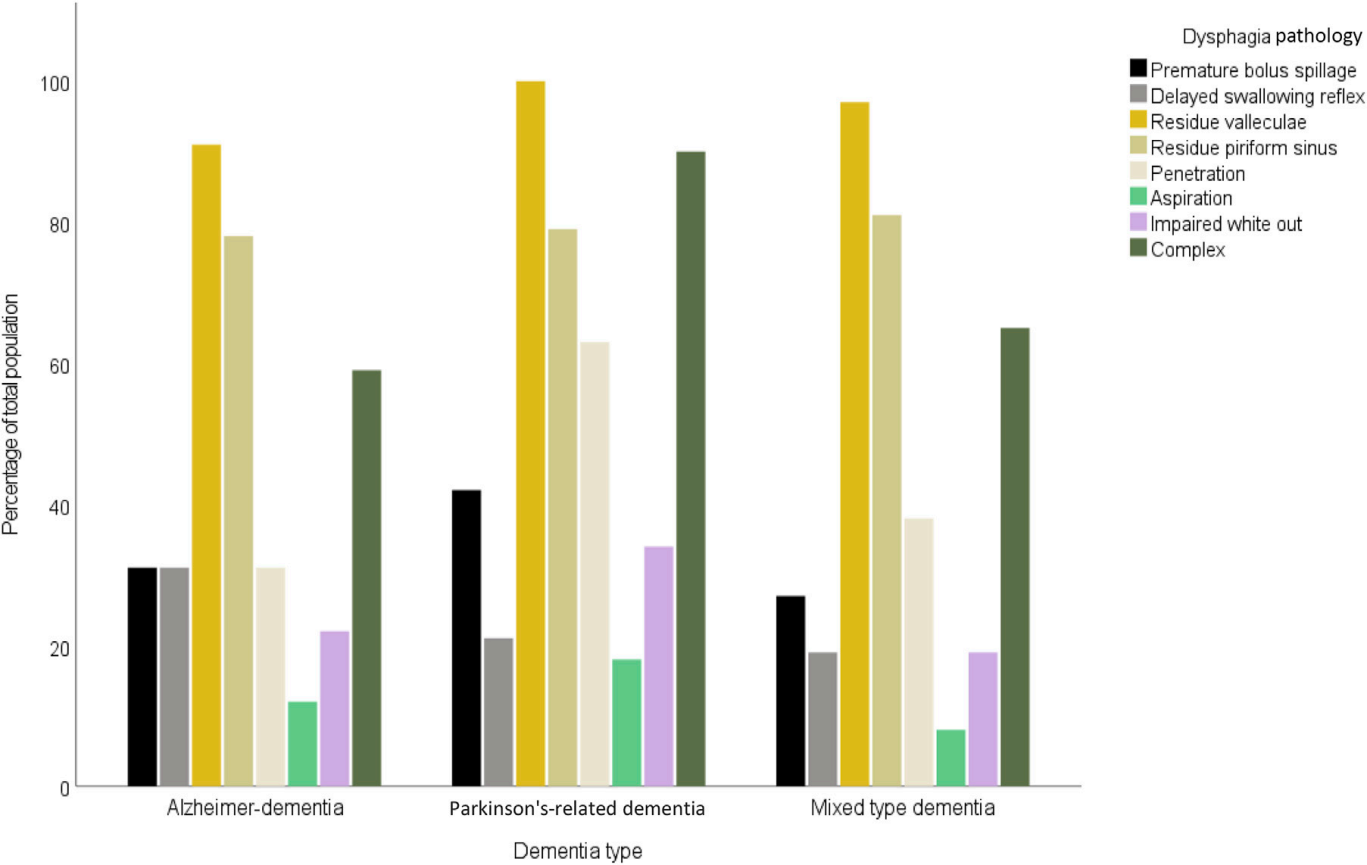
Prevalence of dysphagia pathologies across Alzheimer-dementia, Parkinson-dementia complex, and mixed type dementia.

Table 3 provides a comparative analysis of dysphagia pathologies across three dementia types. Significant variation was noted in the prevalence of penetration in the complex dysphagia pathology. Penetration was significantly more frequent in Parkinson’s-related dementia patients (63%; p = 0.016), compared to Alzheimer’s disease (31%) and mixed-type dementia (38%). Complex dysphagia was also most prevalent in Parkinson’s-related dementia (90%; p < 0.010), and distinctly higher than in Alzheimer’s dementia (59%) and mixed-type dementia (65%). The most common pathology was residue in the valleculae with an equal distribution between dementia types. In contrast, aspiration was least prevalent, with a non-significant variation across types, indicating limited clinical utility for differentiating dementia types. These findings are illustrated by Figures 1, 2. Figure 1 shows the prevalence of the distinct endoscopic findings in the three dementia groups and Figure 2 the same from the perspective of the dementia type.

**TABLE 3:** Comparison of dysphagia pathologies across dementia types.

	Dysphagia pathologies							
	Premature bolus spillage	Delayed swallowing reflex	Residue valleculae	Residue piriform sinus	Penetration	Aspiration	Impaired white out	Complex
Dementia types								
Alzheimer-dementia, n = 32								
**Observed (%)**	**10 (31)**	**10 (31)**	**29 (91)**	**25 (78)**	**10 (31)**	**4 (12)**	**7 (22)**	**19 (59)**
Expected	10.8	7.5	30.8	25.4	14.4	4.2	8.1	23
Parkinson’s-related dementia, n = 38								
**Observed (%)**	**16 (42)**	**8 (21)**	**38 (100)**	**30 (79)**	**24 (63)**	**7 (18)**	**13 (34)**	**34 (90)**
Expected	12.8	8.9	36.6	30.2	17	5	10	27.3
Mixed type dementia, n = 37								
**Observed (%)**	**10 (27)**	**7 (19)**	**36 (97)**	**30 (81)**	**14 (38)**	**3 (8)**	**7 (19)**	**24 (65)**
Expected	12.4	8.6	35.6	29.4	16.6	4.8	9.3	26.6
P value between dementia types	0.363	0.442	0.110	0.951	0.016	0.413	0.273	0.010

Bold values = primary relevance.

Partial correlation analyses indicated a significant association between impaired white out and aspiration among various dysphagia pathologies (r = 0.283, p < 0.001). No other significant correlations were identified between different dysphagia pathologies. In addition, we conducted a regression analysis to investigate the independent effects of various factors, including age, gender, severity of dementia, sarcopenia, handgrip strength, Barthel Index, and frailty, on dysphagia severity. The analysis revealed that none of these risk factors demonstrated a significant association with the severity of dysphagia as evidenced by the following p-values: severity of dementia (p = 0.926), sarcopenia (p = 0.836), age (p = 0.267), gender (p = 0.304), handgrip strength (p = 0.174), Barthel Index (p = 0.609), and frailty (p = 0.737).

## Discussion

The etiology of dysphagia is multifactorial, involving both cognitive impairments associated with dementia and behavioral disorders. These behavioral disorders may include buccofacial apraxia and refusal to eat, resulting in a prolonged oral phase and frontal bolus spillage. Additionally, inter- and post-deglutitive pathomechanistic changes can be observed using FEES. Only few studies have focused exclusively on employing FEES to explore dysphagia in patients with Alzheimer’s disease compared to various stages and forms of dementia (Parlak et al., 2022; Sheikhany et al., 2019; De Stefano et al., 2020; Shapira-Galitz et al., 2019; Suh et al., 2009). This study extends the current understanding by providing insights into the prevalence and characteristics of various dysphagia pathologies across different types of dementia using FEES. Bolus residuals in the vallecula and piriform sinus was detectable in almost all dementia patients (96%). This high prevalence exceeds rates reported in prospective age cohorts, underscoring that dementia-specific neurodegenerative changes significantly contribute to residual function in addition to age-related sensory loss (Labeit et al., 2022).

Our findings highlight significant variation in swallowing dysfunction and findings, with a notably higher prevalence of bolus penetration and complex dysphagia pathology observed in patients with Parkinson’s-related dementia. Our results are consistent with those of a previous study in a relatively large cohort of Parkinson’s disease patients studied with FEES. Similar to our cohort, bolus residues were observed in 93% and bolus penetration was observed in 55% (Pflug et al., 2018). Theories suggest that food residue accumulation, depending of their presence in the valleculae or the sinus piriformis, and bolus penetration could be due to reduced tongue base retraction, velopharyngeal closure, weaker pharyngeal muscles, and a decrease in upper esophageal sphincter opening (Taira et al., 2021; Higo et al., 2001).

Another notable finding of our study is that the most common dysphagia pathology observed across all types of dementia was the presence of residues in the vallecula. In line with our results, bolus residue in the vallecula and the sinus piriformis were also the most prevalent findings in another FEES-based study on Alzheimer’s disease patients (Parlak et al., 2022). Previous research has linked vallecular residues in dementia patients to weakened pharyngeal muscle strength and a delayed swallowing reflex, both of which are associated with an increased risk of aspiration (Shapira-Galitz et al., 2019; Molfenter and Steele, 2013). Contrary to expectations, our results did not indicate a significant association between the presence of residue and an increased rate of aspiration. However, we did observe a correlation between reduced pharyngeal muscle strength, quantified by the white-out phenomenon, and a higher frequency of aspiration in the comparative analysis across groups. The white-out phenomenon has been proposed as a semi-quantitative indicator of pharyngeal contractility (Labeit et al., 2021a) and pharyngeal contractility has been identified as a reliable predictor of aspiration in both FEES and videofluoroscopic swallowing studies (Miles and Hunting, 2023; Ku et al., 2021). This highlights the importance of measuring pharyngeal muscle dynamics as a component of comprehensive dysphagia assessment in dementia patients, providing critical insights into the mechanisms underlying swallowing disorders in this population.

The findings of our study indicate that there are no differences in dysphagia pathologies between patients with Alzheimer’s dementia and those with Alzheimer’s dementia and concomitant small vessel disease. Comparative analyses of these two forms of dementia are lacking in the literature, although an older study employed videofluoroscopy to examine the differences in dysphagia between patients with Alzheimer’s disease and those with vascular dementia (Suh et al., 2009). With regard to the characteristics of dysphagia, no relevant differences were observed in the prevalence of bolus residue in Alzheimer’s patients and patients with vascular dementia (80.0% vs 67.6%, p = 0.502), neither in the occurrence of penetration (73.3% vs. 55.8%, p = 0.248), which is in line with the data in our study. However, in comparison to our cohort, there was a prolongation of the oral phase in patients with Alzheimer’s disease (p = 0.008) and a higher incidence of aspiration in patients with vascular dementia (p = 0.024). It is important to note that the Alzheimer’s patients in the above-cited study showed significantly greater cognitive impairment than those in our cohort. Previous literature has indicated that as dementia progresses, the prevalence of behavioral eating disorders, such as buccofacial apraxia, tends to increase (Seçil et al., 2016). Due to this inter-individual variability of neuropsychiatric symptoms, the reported prevalence of dysphagia in Alzheimer’s disease spans from 2.4% to 100% (Mira et al., 2022).

In summary, no specific endoscopic pathologies of dysphagia could be detected in patients with dementia, except for minor specifics in the Parkinson’s group. The reasons for this may be found in the overlap of the cerebral swallowing network with the neuropathological spread of beta-amyloid pathology in Alzheimer’s disease and the pattern of cerebral microangiopathy. Cortical regions associated both with the swallowing network and the trajectory of neurodegeneration include the lower frontal gyrus, the anterior cingulate cortex, the orbitofrontal cortex, and the supramarginal gyrus (Cheng et al., 2022; Braak and Braak, 1991; Thal et al., 2006). A review of cerebral microangiopathy in patients with dysphagia revealed that white-matter lesions, particularly in the internal capsule, corona radiata, insula, and corpus callosum, were linked to swallowing disorders (Alvar et al., 2021). The lack of differences in the swallowing pathologies of patients in the FEES may be attributed to the fact that Alzheimer’s disease and cerebral microangiopathy progress along a continuum and overlap with areas of the cerebral swallowing network.

For clinical practice, our study shows that patients with dementia have impaired bolus clearance. The relatively rare occurrence of white-out cannot be identified as a reliable indicator of this due to the lack of standardization in recording, evaluation, and interpretation. Individualized dysphagia therapy should therefore continue to be decided on an individual basis for patients with dementia, for example, through meal assistance or careful adjustment of food consistency (Lueg et al., 2024). Our study has several limitations. Conducted as a retrospective analysis with a limited number of participants, generalizability of data is possibly constrained. In addition, the multimorbidity of the patients does not allow for a clear determination of causality between the pathophysiology of dementia and specific types of swallowing disorders. Furthermore, the clinical relevance of dysphagia, particularly in patients with Alzheimer’s disease, is likely influenced by kinematic aspects of swallowing alongside cognitive and behavioral symptoms - factors not fully captured due to the retrospective nature of our study. In future studies, a structured behavioral swallowing assessment, including validated screening for buccofacial apraxia and standardized dysphagia questionnaires administered by speech therapists, would be necessary to differentiate behavioral components of swallowing impairment in dementia. One strength of the study is the utilization of FEES in patients with dementia which is a challenge due to reduced cooperation of the participants. To gain further insight into the influence of dementia on swallowing disorders, it would be beneficial to conduct a comparative analysis with our collective and geriatric patients who have been diagnosed with mild cognitive impairment and Parkinson’s disease without concomitant dementia. Such studies will be important for developing and implementing effective therapies tailored to disease stages and individual dysphagia patterns, focused on enhancing pharyngeal muscle strength and optimizing nutritional interventions.

## Conclusion

Despite small differences in participants with Parkinson’s-related dementia, our study did not identify a typical dysphagia pathology in different types of dementia. Therefore, the individual presentation of dysphagia must be determined in each patient to enable a tailored intervention.

## Data Availability

The raw data supporting the conclusions of this article will be made available by the authors, without undue reservation.

## Supplementary material

SUPPLEMENTARY FIGURE S1 Flowchart of patient selection process for inclusion in the FEES‐based dysphagia analysis.
