# Supplementary figures and images for: Endoscopic characterization of oropharyngeal dysphagia in patients with dementia
Source: Front Aging. 2025 Jun 26;6:1535137. doi: 10.3389/fragi.2025.1535137 (PMC12241064; doi:10.3389/fragi.2025.1535137)

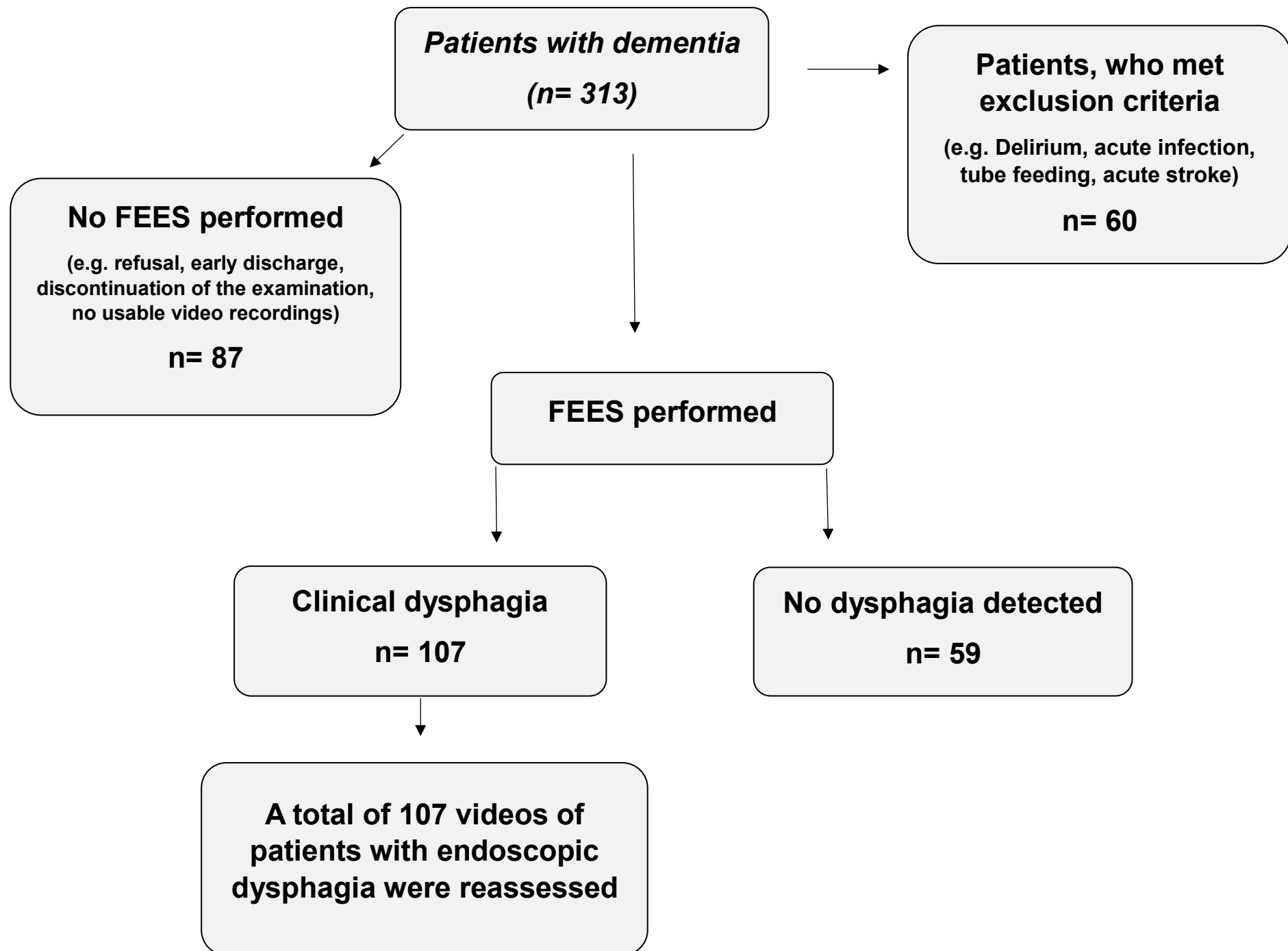

Supplement: Supplementary file 1 [file DataSheet1.pdf]
